# Attitudes towards stress urinary incontinence surgery in Ireland: navigating the pause on mid-urethral sling use

**DOI:** 10.1007/s11845-025-03986-5

**Published:** 2025-06-18

**Authors:** Reut Rotem, Muireann Hickey, Daniel Galvin, Suzanne O’Sullivan, Ciaran Brady, Orfhlaith E. O’Sullivan

**Affiliations:** 1 Department of Urogynaecology, Cork University Maternity Hospital, Cork, Ireland; 2 Department of Obstetrics and Gynecology, Shaare Zedek Medical Center, affiliated with the, Hebrew University School of Medicine, Jerusalem, Israel; 3 Department of Urology, Mercy University Hospital, Cork University Maternity Hospital, Cork, Ireland

**Keywords:** Attitudes, Mesh suspension, Mid-urethral sling (MUS), Stress urinary incontinence (SUI), SUI surgery

## Abstract

**Background:**

Mid-urethral slings (MUS) for the surgical management of stress urinary incontinence (SUI) have been suspended in Ireland since July 2018, significantly impacting treatment options and clinical practice.

**Aims:**

This study aimed to explore the attitudes of consultant obstetricians, gynaecologists, and urologists in Ireland toward SUI surgery following the MUS suspension, including their prior practices, current approaches, and views on MUS safety and potential reinstatement.

**Methods:**

A descriptive, anonymized questionnaire was electronically distributed in early 2023 to consultant members of the Irish Society of Urology and the Continence Foundation of Ireland. Non-consultants, non-medical professionals, and respondents outside Ireland were excluded. Data were analyzed using SPSS v28.

**Results:**

Before the 2018 suspension, 89.5% (17/19) of respondents performed SUI surgeries, with 76.5% (13/17) using MUS-retropubic and 53% (9/17) MUS-transobturator techniques. Post-suspension, 63.2% (12/19) continued performing SUI surgery, primarily urethral bulking (83.3%, 10/12). Regarding safety, 83.3% (15/18) believed MUS led to fewer instances of post-operative voiding dysfunction and 66.7% (12/18) reported fewer complications such as vault prolapse or rectocele. Notably, 44.4% (8/18) had been involved in litigation related to MUS. Despite this, 52.6% (10/19) expressed willingness to resume MUS if the suspension was lifted.

**Conclusions:**

MUS was the preferred SUI procedure prior to suspension due to perceived safety and effectiveness. Over half of consultants surveyed would consider resuming its use, highlighting a need for diverse, evidence-based treatment options and calling for a re-evaluation of the current suspension.

**Supplementary Information:**

The online version contains supplementary material available at 10.1007/s11845-025-03986-5.

## Introduction

Stress urinary incontinence (SUI) represents a significant and prevalent health concern, severely impacting the well-being of individuals [1]. A community-based questionnaire survey in the United Kingdom (UK), conducted in 2014, indicated that upwards of 40% of women aged over 21 years experience some form of urinary incontinence, with SUI being the predominant subtype identified [2]. The management of SUI encompasses a stepwise approach in the form of conservative, medical, and surgical management. The latter includes a broad range of options in its approach, including autologous pubovaginal sling (PVS), urethral bulking, and colposuspension. One of the most commonly employed techniques, however, was the use of synthetic polypropylene mesh as a mid-urethral sling (MUS), which supports the urethra by either a retropubic or transobturator approach [3]. This approach is particularly esteemed for its minimal invasiveness, resulting in reduced operative durations and expedited recoveries compared to more conventional surgical interventions. (i.e. autologous PVS) [4]. The employment of synthetic mesh has been accompanied by a spectrum of complications, including mesh erosion, chronic pain, and dyspareunia [5], engendering considerable patient distress and legal challenges. Consequently, in response to these concerns, the Republic of Ireland instigated a suspension on the utilization of synthetic mesh for the management of SUI and pelvic organ prolapse (POP) in July 2018. In the aftermath, the Health Service Executive (HSE) launched a strategic plan in February 2019, aiming to refine the frameworks surrounding patient consent and the surgical nuances of these procedures [6]. The National Clinical Practice Guideline for the Assessment and Management of Stress Urinary Incontinence in Women, released in December 2022, delineates the current stance on MUS procedures, balancing the suspension with acknowledgment of the technique’s successes and the high satisfaction rates among treated patients [7]. Despite the controversy surrounding MUS in Ireland and the UK, its application remains widespread globally [4, 8–10].

The notion of “suspension” inherently suggests the potential for a future reinstatement of MUS surgical practices. Yet, there is a paucity of research into the disposition of the medical practitioners poised to undertake these procedures once more. This study seeks to elucidate the perspectives and readiness of those practitioners to resume MUS surgeries, thereby contributing valuable insights into the ongoing discourse surrounding this method.

## Material and methods

This study aimed to evaluate the perspectives of consultant obstetricians and gynaecologists, as well as urologists practicing in the Republic of Ireland, on MUS surgery following its suspension in July 2018. The focus was on their experience with mesh-related surgeries, preferred procedures before the suspension, attitudes towards post-operative complications relative to other SUI surgeries, and their likelihood to resume MUS surgery should the suspension be lifted.

### Study design and questionnaire

The study employed a descriptive design using a 31-item questionnaire. The initial questions (1–11) were multiple-choice and gathered participant demographics, including years of practice, fellowship training, and the accrediting institution. Subsequent sections focused on the proportion of clinical practice dedicated to SUI surgery (Questions 12–15), types of surgeries performed before and after the suspension of MUS (Questions 15–18), intentions and preferences for resuming MUS surgery (Questions 19–23), opinions on total mesh excision procedures and the value of multidisciplinary team (MDT) discussions (Questions 24–29), and any involvement in litigation related to MUS surgery (Questions 30–31) (Appendix 1). The questionnaire was developed with input from experienced consultants to align closely with the study’s objectives and was piloted with two consultants: one urogynaecologist and one urologist.

The questionnaire was distributed electronically in early 2023 via email to consultant urologists and urogynaecologists/gynaecologists. Dissemination was facilitated through professional bodies, including the Irish Society of Urology, the Continence Foundation of Ireland, the Urology group of the Royal College of Surgeons in Ireland (RCSI), and the Institute of Obstetricians and Gynaecologists of the Royal College of Physicians of Ireland (RCPI). These bodies represent a broad spectrum of healthcare professionals involved in the management of urinary incontinence, such as nurse practitioners, physiotherapists, and other allied health professionals. Although reminder emails were sent to encourage participation, the exact intervals between reminders were not documented. Due to the electronic nature of the distribution, it was not possible to determine the exact number of individuals who received the questionnaire, and as a result, an accurate response rate could not be calculated.

### Participant selection

Inclusion criteria specified that participants had to be consultant urologists or obstetricians and gynaecologists practicing in the Republic of Ireland. Exclusion criteria included individuals without a medical degree, non-consultant hospital doctors (NCHDs), and those practicing outside of Ireland. Participant anonymity was strictly maintained throughout the study.

### Data analysis

Responses were compiled into a Microsoft Excel spreadsheet stored on a securely locked computer, accessible only to the student investigator and the primary investigator. Data analysis was conducted using the SPSS software (version 28), applying descriptive statistical methods to generate frequency tables and visual representations of the findings. Ethical approval for the study was granted by the Social Research Ethics Committee at University College Cork. The survey began with an information section for participants, with the first question serving as a consent checkbox to ensure informed participation.

## Results

### Participant demographics

Nineteen participants were included: six (31.6%) consultant urologists and 13 (68.4%) consultant obstetricians and gynaecologists’. Most (15, 78.9%) were affiliated with tertiary hospitals, with 11 (64.7%) working in both public and private sectors. A fellowship was completed by 15 (78.9%), primarily in female and reconstructive pelvic surgery (10, 66.7%), with most trained abroad (13, 86.7%). Among them, six (60%) had formal board certification. Experience varied, with five (26.3%) having ≤ 5 years working as a consultant, while 14 (73.7%) had over 10 years, including eight (42.1%) with > 20 years.

### SUI surgeries prior to suspension

Seventeen (89.5%) participants performed SUI surgeries before the July 2018 suspension. Among them, 16 (94.1%) utilized MUS, with MUS-Retropubic performed by 13/17 (76.5%) and MUS-Transobturator by 9/17 (53%). Urethral bulking was performed by 5/17 (29.4%), autologous pubovaginal sling (PVS) by 2/17 (11.8%), and colposuspension by 1/17 (5.9%).

**Fig. 1:**
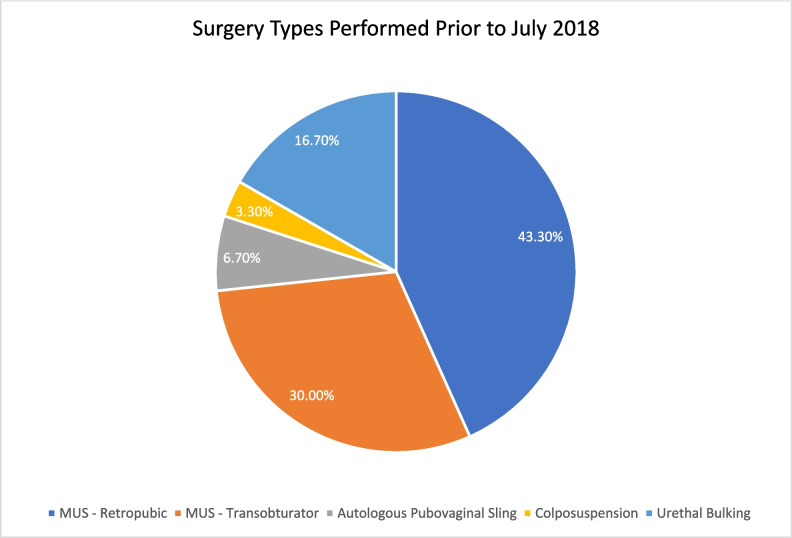
The proportion of surgery types performed prior to the suspension of MUS surgery in July 2018

In terms of technique variety, 8/17 (47.0%) used only one method, 5/17 (29.4%) used two, 3/17 (17.6%) used three, and 1/17 (5.9%) employed four. None performed all five types of surgery. All consultants utilizing three or more methods were fellowship-trained in female and reconstructive pelvic surgery. MUS techniques (retropubic and transobturator) comprised 73.3% of all SUI surgeries performed before the suspension (Fig. 1).

**Fig. 2:**
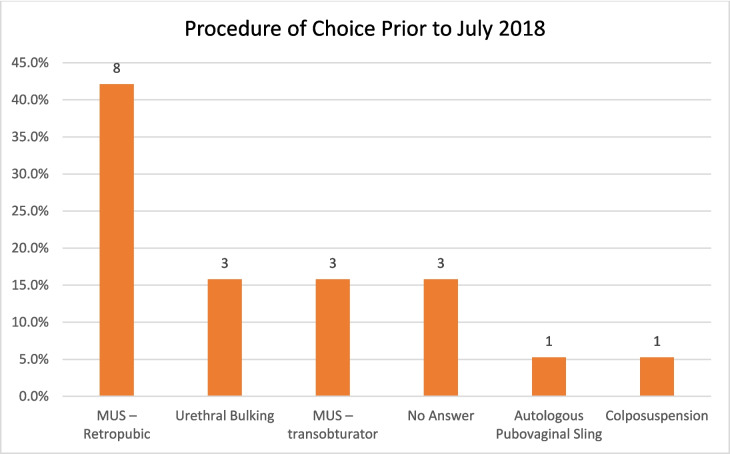
The procedure of choice of those performing SUI surgery prior to July 2018

When asked about their preferred procedure before the suspension, MUS-Retropubic was most common (9/17, 52.9%), followed by both MUS-Obturator and urethral bulking (3/17, 17.6% each). One consultant (5.9%) preferred colposuspension, and another selected autologous fascial sling (Fig. 2).

### SUI surgeries post-suspension

Following the suspension, 12/19 (63.2%) continued performing SUI surgeries. Urethral bulking was the predominant procedure (10/12, 83.3%), reflecting a notable increase from pre-suspension rates. PVS was performed by 7/12 (58.3%), and colposuspension by 2/12 (16.7%).

### Likelihood of returning to MUS surgery

If the suspension were lifted, 10/19 (52.6%) expressed willingness to resume MUS surgery, 5/19 (26.3%) were against it, and four were either unsure or did not respond. All those in favour preferred the retropubic approach.

### Safety profile of SUI surgeries

**Fig. 3:**
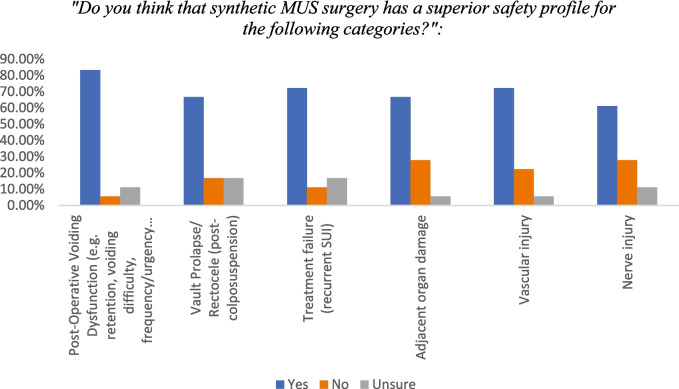
Comparison of the safety profile of MUS surgery to other SUI surgical procedures

All participants (100%) acknowledged that SUI surgeries carry risks, with complication rates varying by procedure. A majority (15/18, 83.3%) believed MUS was associated with lower rates of post-operative voiding dysfunction compared to alternative surgeries (Fig. 3).

Regarding complications, 12/18 (66.7%) felt MUS resulted in fewer cases of vault prolapse or rectocele, particularly compared to colposuspension. Similarly, 12/18 (66.7%) considered MUS to cause less collateral damage than autologous slings or colposuspension, though bulking agents were perceived as the least invasive. Additionally, 13/18 (72.2%) reported that MUS had a lower risk of vascular injury, and 11/18 (61.1%) believed it carried a reduced risk of nerve injury. Collectively, these findings suggest that participants favoured MUS for its perceived safety and effectiveness.

### Mesh excision and litigation

Regarding total mesh excision (TME) for managing mesh erosions, 13/18 (72.2%) believed it should rarely be performed, while 17/18 (94.4%) felt it could worsen symptoms in some patients. Most respondents (16/18, 88.9%) favoured local excision over TME for vaginal mesh erosions.

Five (27.8%) respondents indicated a need for additional training, supervision, or mentorship for performing major alternative SUI surgeries, such as colposuspension or autologous PVS.

Regarding multidisciplinary team (MDT) discussions, 5/18 (27.8%) believed SUI surgeries should involve MDT input, including surgeons, physiotherapists, and nurses. A majority (11/18, 61.1%) reported access to a local incontinence MDT.

On litigation, 8/18 (44.4%) had been involved in legal cases related to MUS surgery, while 3/18 (16.7%) had provided medico-legal advice to claimants.

## Discussion

In this pioneering study, we explored the perspectives of healthcare professionals in Ireland regarding MUS surgery since its suspension in 2018, aiming to shed light on future urogynaecological practices. A significant majority of participants reported performing SUI surgeries before the suspension, with 73.3% of cases using either the MUS-Retropubic or MUS-Transobturator approaches. This preference underscores the central role MUS played in SUI management prior to the suspension, reflecting its perceived benefits, such as efficiency and patient recovery [4].

Studies in Ireland and in the UK have showed a downwards trend in SUI surgery over all since the suspension of MUS procedures [11, 12]. The study by McCraith et al. [12] provides valuable insight into this trend, showing a notable reduction in the number of SUI surgical interventions post-suspension. Despite this decrease, the study also highlights that when SUI surgeries were performed, MUS techniques remained a preferred option among those practitioners who continued to operate. This aligns with our own findings, which reflect a strong preference for MUS among respondents, even in the face of regulatory restrictions. These observations point to a broader issue: The decline in surgical intervention for SUI may signal an unmet need for effective treatment options, leaving many patients without optimal care.

Following the suspension of MUS, a clear shift towards alternative procedures such as urethral bulking, autologous PVS, and colposuspension was observed, with urethral bulking emerging as the most commonly performed procedure. This pivot, despite urethral bulking's well-documented lower efficacy [13], underscores the unintended consequences of regulatory constraints on the quality of patient care. Practitioners have been compelled to adopt fewer effective treatments due to the limited surgical options available. Our survey also highlighted the need for additional training in these alternative methods, reflecting the importance of upskilling clinicians to ensure they can provide high-quality care in the absence of MUS.

Despite a significant number of respondents (44.4%) having been involved in litigation related to MUS surgery, our findings revealed a strong willingness among practitioners to resume its use, with 52.6% expressing readiness to perform MUS again if the suspension is lifted. This underscores the unwavering confidence these clinicians have in the procedure’s effectiveness; despite the legal challenges they have faced. Such strong belief in MUS’s benefits speaks to its perceived value in managing SUI effectively.

It is important to acknowledge that no surgical procedure is without complications, but when evaluating the safety of MUS, it must be viewed from a broader perspective. This includes considering how invasive the alternatives are, the ease and availability of other options, and the overall frequency of complications. While it is clear that, in the event that a complication occurs, it can be highly debilitating, these events are not common [14, 15] and should be framed appropriately within the broader discussion of risks. MUS continues to stand out as a procedure with a manageable safety profile, especially when weighed against the invasiveness and challenges of the alternatives [16]. Recent long-term data further support this, showing that even after 20 years, most women remained satisfied with their MUS procedure and would choose the mesh tape again if facing the decision today [17].

It is crucial that all risks associated with the use of mesh, as well as its alternatives, are transparently presented to patients, allowing them to fully understand the potential benefits and drawbacks of each option. Patients should be empowered through autonomy and shared decision-making to determine the most suitable treatment for their condition, rather than having these decisions directed by healthcare providers or regulatory bodies without offering them a choice. The current suspension in the UK and Ireland limits this autonomy, as it restricts access to a widely used and globally accepted procedure. Women in Ireland and the UK should not be deprived of the same treatment options available to women worldwide, especially when mesh procedures continue to be successfully performed in many other countries. By withholding this option, we risk disempowering patients, diminishing their ability to engage in informed, collaborative decision-making about their own healthcare. The ability to choose, based on a comprehensive understanding of both the risks and benefits, is a cornerstone of patient-centred care that must be preserved.

It is important to recognize the substantial efforts being made within Ireland by healthcare professionals to advocate for the resumption of MUS as a treatment option. Ongoing debates and multidisciplinary discussions have taken place, with clinicians striving to overcome the regulatory and clinical constraints that have followed the suspension of MUS. A national recovery plan aimed at facilitating the surgical reintroduction of MUS has been developed, incorporating input from urogynaecologists, urologists, policymakers, and patient advocacy groups [18]. However, despite these efforts, no significant progress has been made, and the suspension remains in effect, with no indication that it will be lifted in the foreseeable future. The ongoing pause in MUS availability continues to limit treatment options for women in Ireland, leaving both patients and clinicians navigating alternative methods that may not offer the same efficacy or convenience. The absence of a clear path forward not only highlights the complexity of balancing patient safety with surgical innovation but also underscores the challenges faced in reinstating a procedure that remains in use globally.

### Strengths and limitations

A major strength of this study lies in its novelty, as it is the first in Ireland to explore healthcare professionals’ attitudes towards MUS surgery post-suspension and assess the readiness for its potential reinstatement. It complements existing research by providing an extended view into the future outlook on SUI surgery preferences and safety perceptions within the Irish context.

However, the study is not without limitations. The small sample size, which was restricted to consultants, may not fully capture the broader perspectives within the field, particularly those of NCHDs, who represent the future of urogynaecology. While the insights of NCHDs could be invaluable, their exclusion was deemed necessary due to the study's focus on current practices and decision-making authority. Furthermore, we were unable to characterize those who chose not to participate in the study, which may introduce selection bias. This lack of information on non-respondents makes it difficult to determine whether their views and experiences differ significantly from those of the participants, potentially affecting the generalizability of our findings.

## Conclusion

As the field of urogynaecological care continues to evolve, it is essential that SUI management remains aligned with the principles of efficacy, safety, and patient preference. While this study underscores the historical importance of MUS surgery in treating SUI, it also reveals a cautious willingness among practitioners to consider its reintroduction, provided that patient safety and informed consent are prioritized. The findings emphasize the need for patient-centred decision-making, ensuring that all available treatment options, including alternatives to MUS, are thoroughly discussed so that patients can make well-informed choices that best suit their individual needs.

## Supplementary Information

Below is the link to the electronic supplementary material.

ESM 1 (DOCX 520KB)

## Data Availability

The data that support the findings of this study are available from the corresponding author upon reasonable request.
