# Supplementary figures and images for: Attitudes towards stress urinary incontinence surgery in Ireland: navigating the pause on mid-urethral sling use
Source: Ir J Med Sci. 2025 Jun 18;194(4):1485–91. doi: 10.1007/s11845-025-03986-5 (PMC12413413; doi:10.1007/s11845-025-03986-5)

***Appendix 1 – Questionnaire***

**
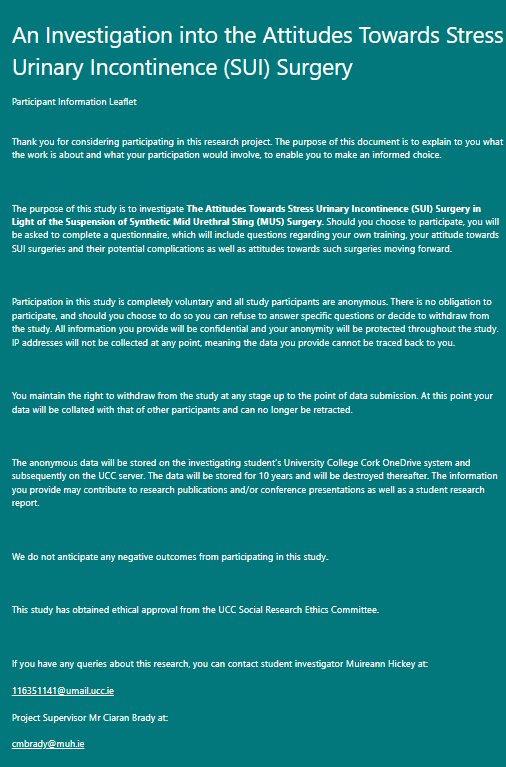
**

**
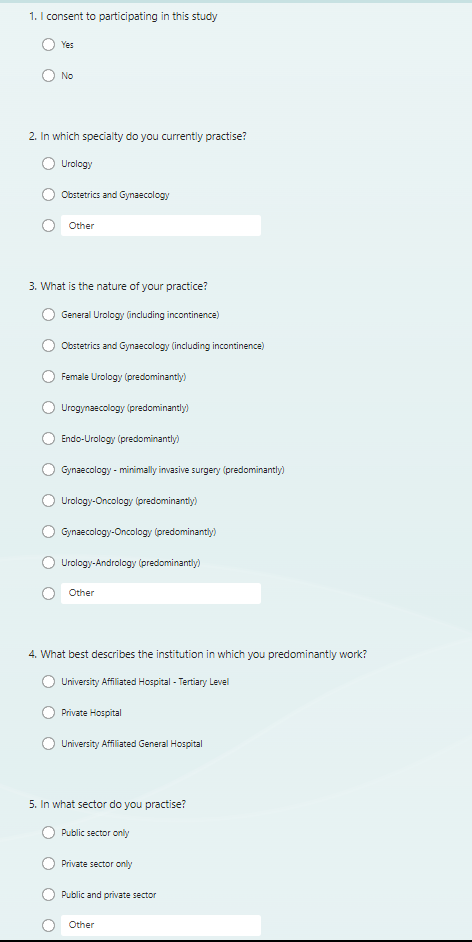
**

**
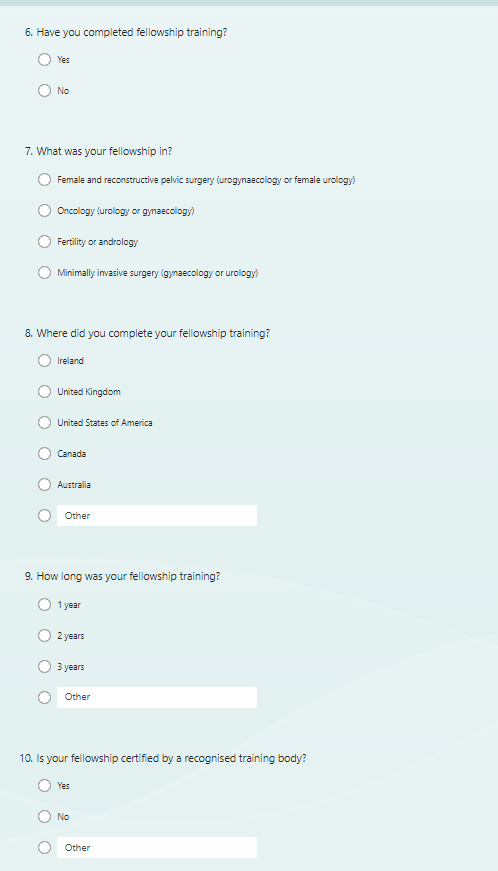
**

**
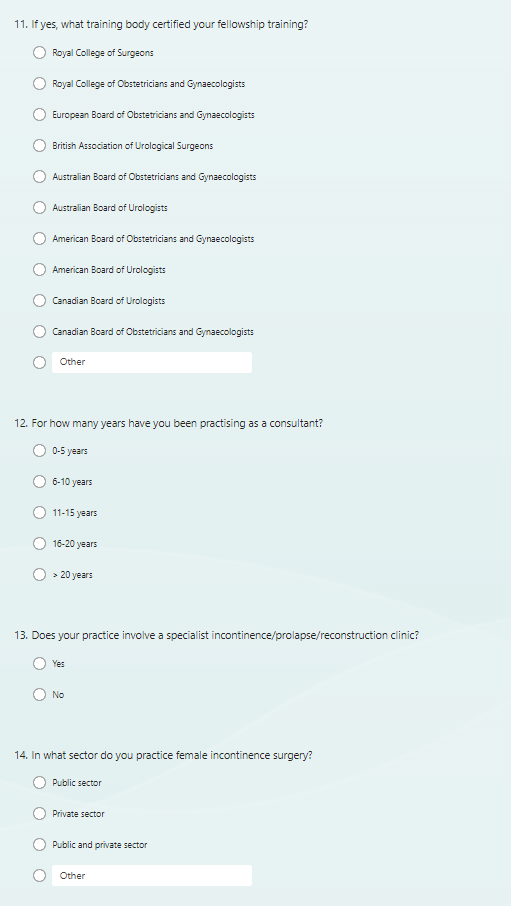
**

**
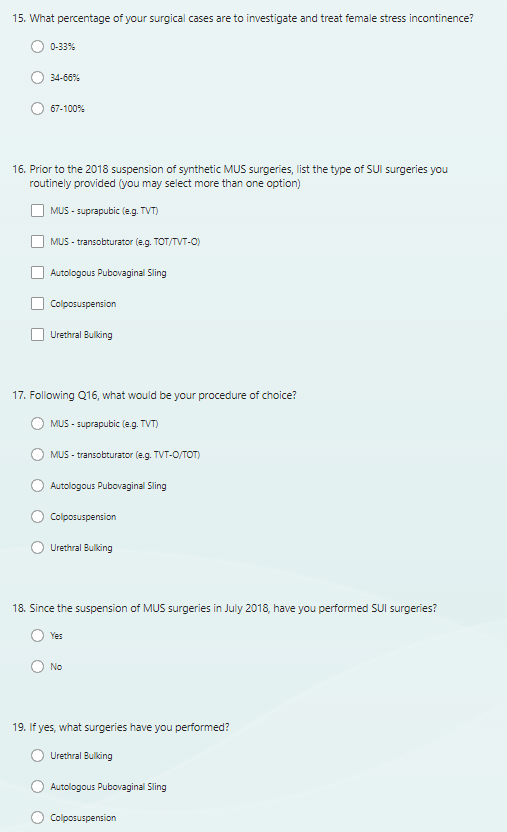
**

**
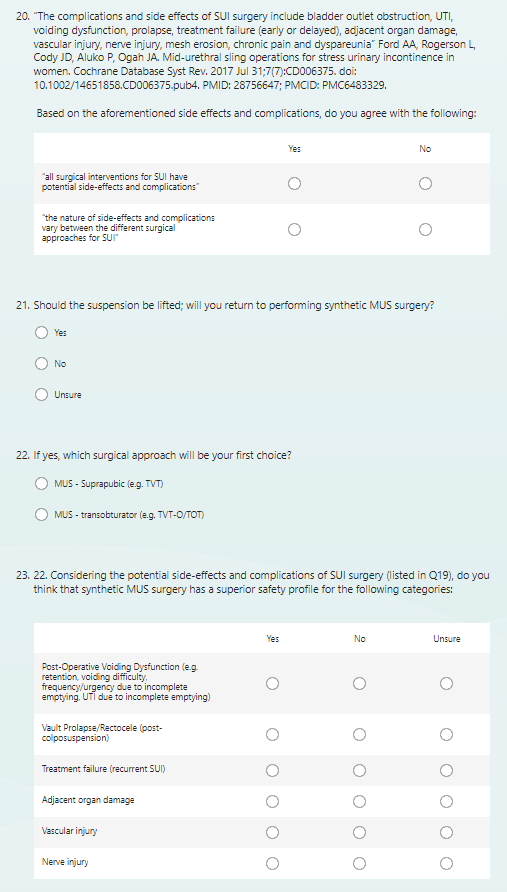
**

**
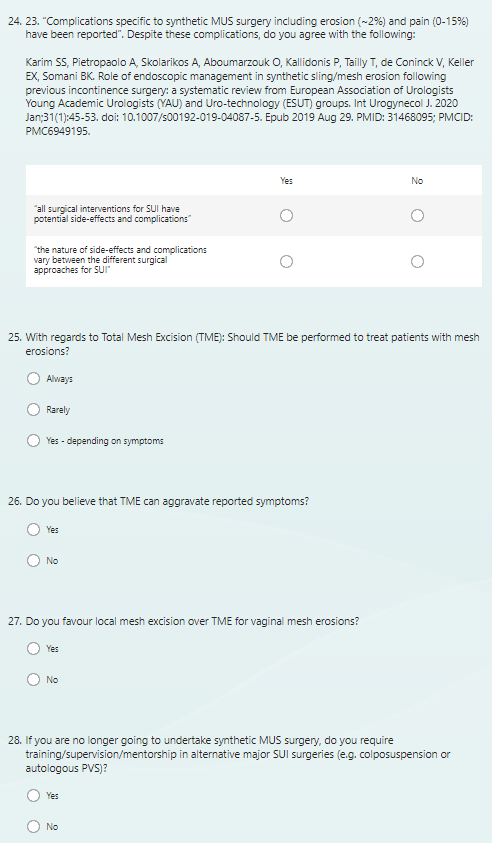
**

**
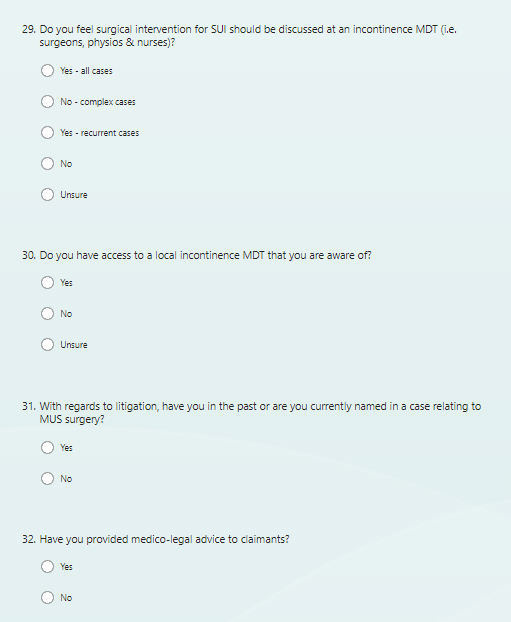
**

Supplement: Supplementary file 1 — (DOCX 520KB) [file 11845_2025_3986_MOESM1_ESM.docx]
